# Identification of a Novel Immune-Related Prognostic Biomarker and Small-Molecule Drugs in Clear Cell Renal Cell Carcinoma (ccRCC) by a Merged Microarray-Acquired Dataset and TCGA Database

**DOI:** 10.3389/fgene.2020.00810

**Published:** 2020-08-18

**Authors:** Guan-Fa Xiao, Xin Yan, Zhao Chen, Ren-Jie Zhang, Tong-Zu Liu, Wan-Li Hu

**Affiliations:** ^1^Department of Urology, Zhongnan Hospital of Wuhan University, Wuhan, China; ^2^Department of Pediatric surgery, Ganzhou Maternal and Child Health Hospital, Ganzhou, China

**Keywords:** CTLA4, clear cell renal cell carcinoma, immune-related prognostic biomarkers, immune infiltration, small molecule drugs

## Abstract

Clear cell renal cell carcinoma (ccRCC) is one of the most common histological subtypes of renal cancer, with a poor prognosis. Our study aimed to identify a biomarker that is significantly associated with ccRCC prognosis and novel immunotherapeutic targets, as well as some novel molecular drugs for ccRCC. Based on the overlap of The Cancer Genome Atlas (TCGA)-Kidney Renal Clear Cell Carcinoma (KIRC) data and the ImmPort database, we obtained 1,292 immune-related genes (IRGs) and constructed a weighed co-expression network based on the IRGs. A total of 39 hub genes were screened out in three modules. CTLA4, which had the highest connectivity degree among the screened genes in a protein–protein interaction network (degree = 24), was selected. Internal validation based on the GEPIA database revealed that patients with a higher expression of CTLA4 had a significantly shorter overall survival time and disease-free survival time. Expression of CTLA4 was also closely correlated with local recurrence, pathologic stage, and immune infiltration level. External validation based on the Oncomine database and merged microarray-acquired dataset validated the mRNA expression level of hub genes. Gene-set enrichment analysis revealed that six KEGG signaling pathways, which were significantly associated with CTLA4, were enriched on immune-related pathways. Further analysis according to the TIMER database demonstrated that CTLA4 expression was positively related to dendritic cells (cor = 0.446, *P* = 1.32E-23) and negatively associated with tumor purity (cor = −0.267, *P* = 5.51E-09). Finally, we screened out 293 differentially expressed genes by integrating six datasets from the GEO database. The Connectivity Map (CMap) analysis revealed the strong potential of three small molecule drugs (monensin, quercetin, and fenbufen) for ccRCC treatment. In conclusion, CTLA4 was identified and validated in prognosis of ccRCC. CTLA4 may be a new prognostic biomarker and immunotherapeutic target for ccRCC. Monensin, quercetin, and fenbufen may be novel choices for ccRCC treatment.

## Introduction

Immunotherapy for cancer dates back to the late 19th century, when Dr. William Coley injected live bacteria into tumors and then successfully treated hundreds of cancer patients with bacterial “toxins” (Marabelle et al., 2017). Nowadays, with the clinical successes of immune-checkpoint blockade and chimeric antigen receptor T cell therapies, immunotherapy has again become the focus of cancer treatment.

Wang et al. (2019) suggested that checkpoint-related proteins may be associated with advanced disease, recurrence, and survival in patients with clear cell renal cell carcinoma (ccRCC). Hassler et al. (2019) demonstrated that monoclonal antibodies targeting immune-checkpoint inhibitors have antitumor effects on metastatic renal cancer. Immunotherapy has demonstrated an optimistic therapeutic effect on renal cancer (Fong et al., 2019; Muto and Gridelli, 2019) and has become a hot topic in the treatment of renal cancer. The search for immune prognostic biomarkers associated with ccRCC may lead to new treatments.

Renal cancer is among the 10 most common cancers in western countries, representing 3–5% of all cancers (Siegel et al., 2018). RCC accounts for approximately 90% of all renal cancers, most of which (80–90%) are ccRCC (Ljungberg et al., 2015). Biomarkers for the early diagnosis and follow-up of RCC are still unavailable. More than 50% of RCCs are detected incidentally, and approximately 30% of RCC patients have developed metastases when diagnosed. Moreover, 30–50% of RCC patients develop metastases during follow-up (Rydzanicz et al., 2013). The prognosis of ccRCC is extremely poor, and there is no effective prognostic marker. The identification of novel prognostic biomarkers that might be targets for immunotherapy is crucial.

The small sample size of the ccRCC datasets from the Gene Expression Omnibus (GEO) database might lead to random and unreliable results. Thus, in the present study we integrated six data sets for screening differentially expressed genes (DEGs), identifying model drugs, and verifying immune-related biomarkers. Initially, 1,292 immune-related genes (IRGs) were screened out by overlapping data from The Cancer Genome Atlas-Kidney Renal Clear Cell Carcinoma (TCGA-KIRC) and the ImmPort databases. Based on these IRGs, we constructed a weighed co-expression network and a protein–protein interaction (PPI) network and selected CTLA4. Further analyses explored the potential values of CTLA4 through external and internal validation. CTLA4 was closely correlated with overall survival (OS), disease-free survival (DFS), local recurrence, pathologic stage, and immune infiltration level of patients with ccRCC. Finally, three molecular drugs were screened for the treatment of ccRCC based on the 293 DEGs obtained by integrating six data sets from GEO database and CMap analysis.

The study findings identified and validated CTLA4 in prognosis of ccRCC. CTLA4 might be a new prognostic biomarker and immunotherapeutic target for ccRCC. The three small molecular drugs that were screened (monensin, quercetin, and fenbufen) might be novel choices for ccRCC treatment.

## Materials and Methods

### Data Collection and Preprocessing

**FIGURE 1:**
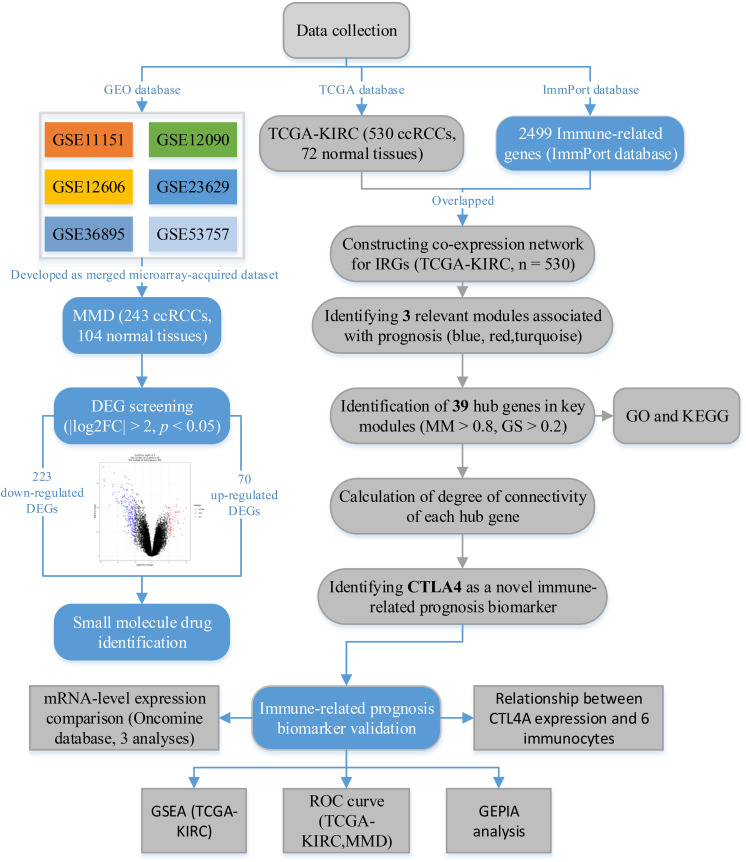
The flow diagram of this study. Data preparation, analysis, and validation are shown in the flow diagram.

A flow diagram of the data preparation, processing, analysis, and validation is shown in Figure 1. We first downloaded six independent GEO datasets (GSE53757, GSE11151, GSE12090, GSE12606, GSE23629, and GSE36895) as the raw data from the GEO database^1^. All six GEO datasets were profiled on the GPL570 platform, which were first Robust Multichip Average (RMA)-normalized using the R package “affy” (Gautier et al., 2004). Next, we generated a unified, ccRCC-specific, merged microarray-acquired dataset (MMD) by preprocessing, merging, and ComBat-adjusting the six datasets using the *in silico* merging package (Taminau et al., 2012) in R software. Finally, probes were annotated using the GPL570 annotation files. A total of 243 ccRCC samples and 104 normal kidney tissues were included in this study.

Clear cell renal cell carcinoma microarray data (TCGA-KIRC data), displayed as count number, were downloaded from the TCGA database^2^. After excluding unqualified samples, a total of 530 ccRCC samples and 72 normal samples were used in this study. The samples contained complete clinical information including OS time and OS status (including age, gender, laterality, and pathologic stage). TCGA-KIRC data displayed as count number, and normalized and log2 transformations were conducted, relying on the R package “DEseq.2” (Anders and Huber, 2010).

A comprehensive list of IRGs that included 2,499 genes was retrieved from the ImmPort database^3^. The 1,292 genes that overlapped between IRGs and the gene list of the TCGA-KIRC data were chosen for subsequent analysis.

### Weighted Co-expression Network Construction

The “WGCNA” package in R software was used to construct a weighed co-expression network based on IRGs. First, the expression data profile of IRGs was tested to check if they were good samples or good genes by two independent methods (goodSamplesGenes [gsg] method and sample network method). Specifically, the Euclidean distance-based sample network is simply the canonical Euclidean distance-based network *A(uv)* = *1-| |S(u)-S(v)| | ^2/maxDiss*. Next, we use the standardized connectivity *Z.ku* = [*ku-mean(k)*]/[*sqrt*(*var*(*k*))] to identify array outliers. Samples with Z.Ku < −2.5 were regarded as outlying samples, which were removed from WGCNA. Then, a weighted adjacency matrix was constructed using the power function: *aij* = *|sij| ^β^* (*sij* = the absolute value of the Pearson correlation coefficient between gene i and gene j; *aij* = adjacency between gene i and gene j; β is a soft-thresholding parameter that emphasizes high correlations at the expense of low correlations). Here, the power of β = 4 (scale free *R*^2^ = 0.84, Supplementary Figure S1) was selected to ensure a scale-free network. Subsequently, the adjacency was transformed into a topological overlap matrix (TOM) and the corresponding dissimilarity (1-TOM) was also calculated. In this study, we classified genes into gene modules by applying branch-cutting methods with parameters set as follows: minClusterSize = 30 and deepSplit = 2. Moreover, we merged some highly similar modules (correlation ≥0.75) and a multidimensional scaling (MDS) plot was plotted to estimate the bio-similarity of modules. Finally, the gene network was visualized with all the genes.

### Identification of Relevant Modules

After relating modules to clinical traits, the Module Significance (MS), the correlation between the module eigengene and the trait, was calculated. In general, the higher the value of MS, the more important is the module. This study focused on the pathologic stage, which was regarded as the most important clinical trait. We regarded gene modules with the top three | MS| as relevant modules. Gene Significance (GS, the correlation between the gene and the trait) and Module Membership (MM, the correlation between the gene expression profile and the module eigengene) were also calculated. In WGCNA, the gray module contained a set of unassigned genes that did not belong to any of the modules, which was removed for subsequent analysis.

### Hub Gene Identification

In this study, hub genes in the three relevant modules were defined by |MM| > 0.8 and |GS| > 0.2, which were regarded as hub genes in the co-expression network. Furthermore, all the hub genes in the co-expression network were uploaded to the Search Tool for the Retrieval of Interacting Genes (STRING) database^4^ (Szklarczyk et al., 2017) to construct a PPI network. Parameters for the PPI network were set as follows: network scoring: degree cutoff = 2; cluster finding: node score cutoff = 0.2, k-core = 2, and max. depth = 100. The degree of connectivity of each gene was calculated by a tool in Cytoscape (network analyzer). The gene with the highest degree of connectivity was defined as hub gene in the PPI network, which was also regarded as the prognostic biomarker in this study.

### Functional and Pathway Enrichment Analysis

To explore potential functions of hub genes in relevant modules, Gene Ontology (GO) enrichment analysis and Kyoto Encyclopedia of Genes and Genomes (KEGG) (Kanehisa and Goto, 2000) pathway analysis were performed through the “clusterProfiler” (Yu et al., 2012) in R software. Gene sets and KEGG signaling pathways were regarded as significantly enriched gene sets when *P* < 0.05.

### Gene Expression Profiling Interactive Analysis (GEPIA)

To explore the association between hub gene and prognosis of ccRCC, we analyzed two survival types, OS and disease-free survival (DFS), based on the GEPIA webtool (Tang et al., 2017)^5^. Moreover, we compared the expression levels of hub genes between ccRCC tissue and normal tissue as an internal validation. Unpaired *t* test was used for statistical significance measuring. In addition, we also explored the expression difference between different stages (I,II,III, and IV). Statistical significance was measured by one-way analysis of variance (ANOVA).

### Validation of Hub Genes According to mRNA Expression Level

After the use of GEPIA, we assessed the mRNA expression levels of hub genes in ccRCC tissue and normal tissue based on the Oncomine database (Rhodes et al., 2004)^6^ for external validation. Additionally, the MMD was used to validate the mRNA expression levels of hub genes. Student’s *t* test was used to measure the statistical significance.

### Prognostic Value of Hub Gene Validation

Using “plotROC” in R software, receiver operating characteristic (ROC) curves were drawn based on the TCGA-KIRC and MMD large datasets. The area under the curve (AUC) was calculated to distinguish ccRCC samples from normal tissues. Hub genes were concluded to have important prognostic value and diagnostic value when the AUC of a hub gene was ≤0.75.

### Exploring Relationship Between Hub Gene Expression and Immunocytes

Based on TIMER (Li et al., 2017)^7^, we investigated the correlation between hub genes expression and immunocytes. Six tumor-infiltrating immune cell types (B cells, CD8 + T cells, CD4 + T cells, macrophages, neutrophils, and dendritic cells) were included for this analysis (Li et al., 2016). Hub genes were considered highly correlated with an infiltrating level of an immunocyte when |correlation coefficient (cor)| ≥0.2 and *P* value < 0.05.

### Gene-Set Enrichment Analysis (GSEA)

To identify the potential functions of hub genes, GSEA (Subramanian et al., 2005)^8^ was conducted for detecting whether a series of priori defined biological processes (BPs) were enriched in the gene rank derived from DEGs. Annotated gene sets “c2.cp.kegg.v7.0.symbols.gmt” were chosen as the reference gene sets. Nominal *P* < 0.05, |ES| > 0.6, gene size ≥100, and FDR < 0.05 were chosen as the cutoff criteria in this study.

### DEG Screening

In addition to identification of an immune-related prognostic biomarker, we also aimed to screen out some small molecule drugs for ccRCC treatment. Hence, we first identified DEGs between normal tissues and ccRCC tissues using the “Limma” (Ritchie et al., 2015) package in R software. Genes with an adjusted *P* < 0.05 and |log2 fold change (FC)| ≥2.0 were regarded as DEGs.

### Molecule Drug Identification

After screening out DEGs, based on these DEGs, we performed Connectivity map (CMap) analysis (Lamb et al., 2006) to explore molecule drugs. Correlations between drugs and ccRCC were sorted by the absolute value of enrichment. The top three drugs were regarded as having potential value for ccRCC treatment.

## Results

### Weighted Co-expression Network Construction and Identification of Relevant Modules

**FIGURE 2:**
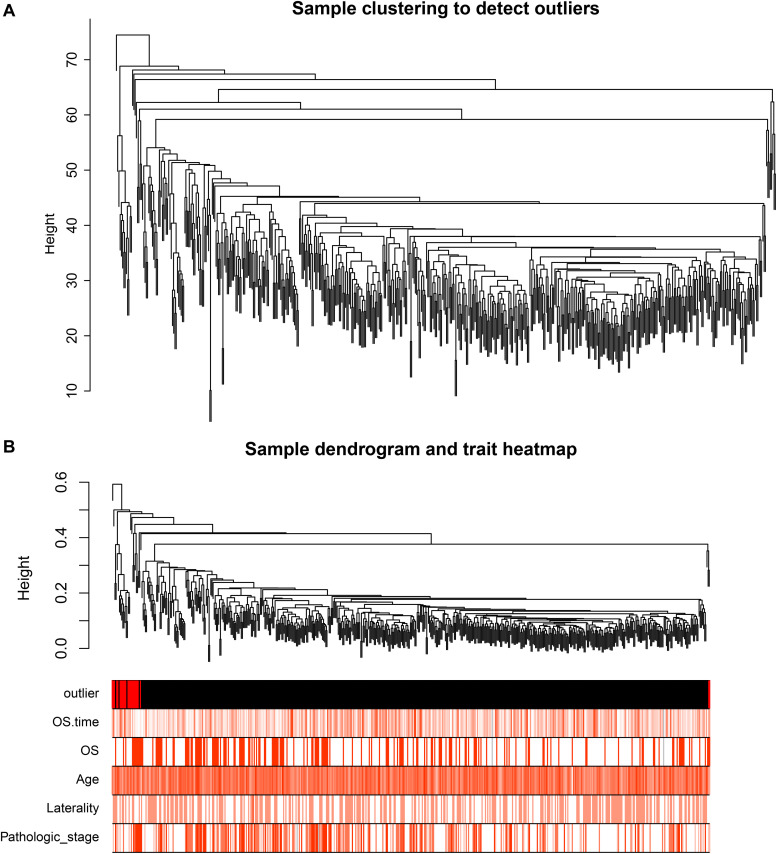
**(A)** Sample clustering to detect outliers. **(B)** Sample dendrogram and trait heat map. The color intensity was proportional to older age, OS (overall survival) time, OS, gender, laterality, and pathologic stage.

**FIGURE 3:**
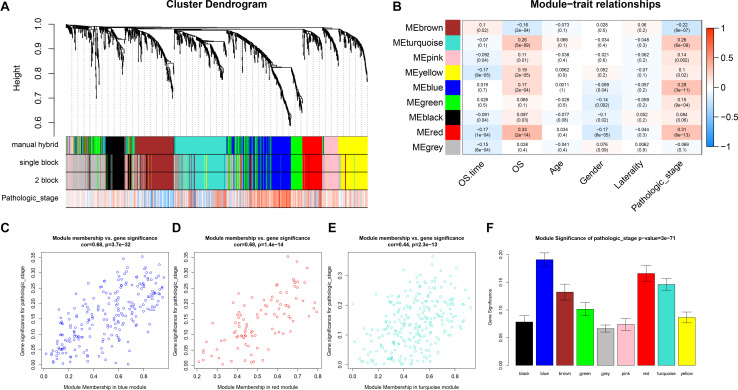
Identification of relevant modules associated with clinical information. **(A)** Dendrogram of all differentially expressed genes clustered based on a dissimilarity measure (1-TOM). **(B)** Heat map of the correlation between module eigengenes and different clinical information of ccRCC (OS time (overall survival time), OS (overall survival status, age, gender, laterality, and pathologic stage). **(C)** Scatter plot for correlation between gene module membership in the blue module (pathologic stage) and gene significance. **(D)** Scatter plot of MEs in red module. **(E)** Scatter plot of MEs in turquoise module. **(F)** Distribution of average gene significance and errors in the modules associated with the progression of ccRCC.

**FIGURE 4:**
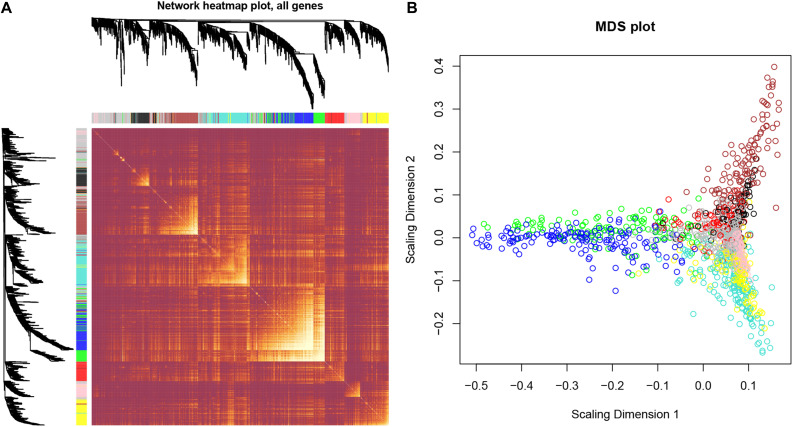
Interaction relationship analysis of co-expression genes and construction of a classical MDS plot. **(A)** Different colors of horizontal axis and vertical axis represent different modules. The brightness of yellow in the middle represents the degree of connectivity of different modules. There was no significant difference in interactions among different modules, indicating a high-scale independence degree among these modules. **(B)** Classical MDS plot whose input is the TOM dissimilarity. Each dot (gene) is colored by the module assignment.

After identifying outlier samples, totally 23 samples were removed from further analysis (Figure 2). Based on IRGs, the “WGCNA” package in R software was used to construct a weighed co-expression network. A total of eight modules was identified (Figure 3A). The pathologic stage was chosen as the clinical information of interest. Based on the relation of modules to clinical traits, we found that the module eigengene denoted in blue, red, and turquoise in Figure 3B was highly correlated with pathologic stage compared to the other modules. By comparing the Module Significance, we determined that the MS denoted as the blue, red, and turquoise modules in Figure 3F was higher than in other modules. We regarded these modules as relevant modules. Figures 3C–E illustrate the correlation between MM and GS in blue, red, and turquoise, respectively. A network heat map and a classical MDS plot was created (Figures 4A,B).

### Hub Gene Identification

**FIGURE 5:**
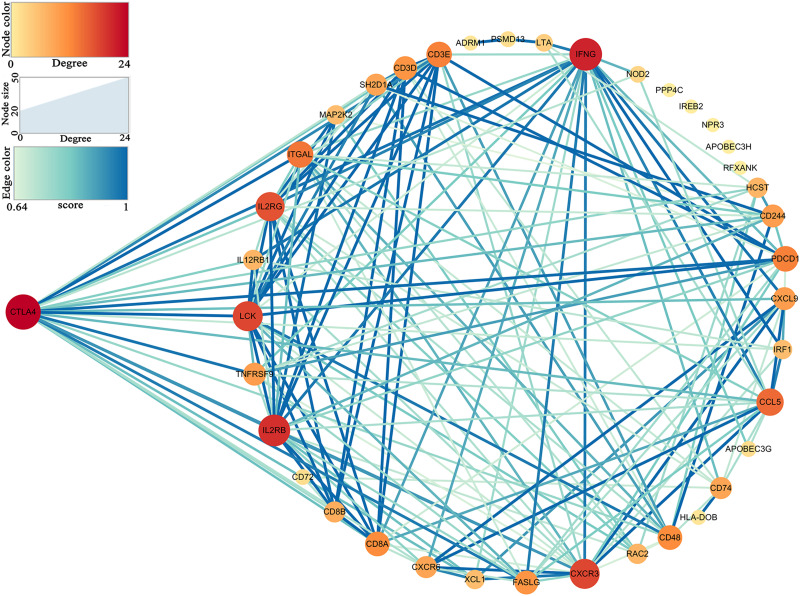
Protein–protein interaction (PPI) network of hub genes in key modules.

Thirty-nine hub genes were screened out by |MM| > 0.8 and |GS| > 0.2 in the three aforementioned relevant modules; CTLA4 displayed the highest connectivity degree (degree = 24) among these genes (Supplementary Table S1). The constructed PPI network also revealed that CTLA4 has the highest degree of connectivity (Figure 5). Therefore, CTLA4 was chosen as the candidate gene for further validation.

### Functional and Pathway Enrichment Analysis

**FIGURE 6:**
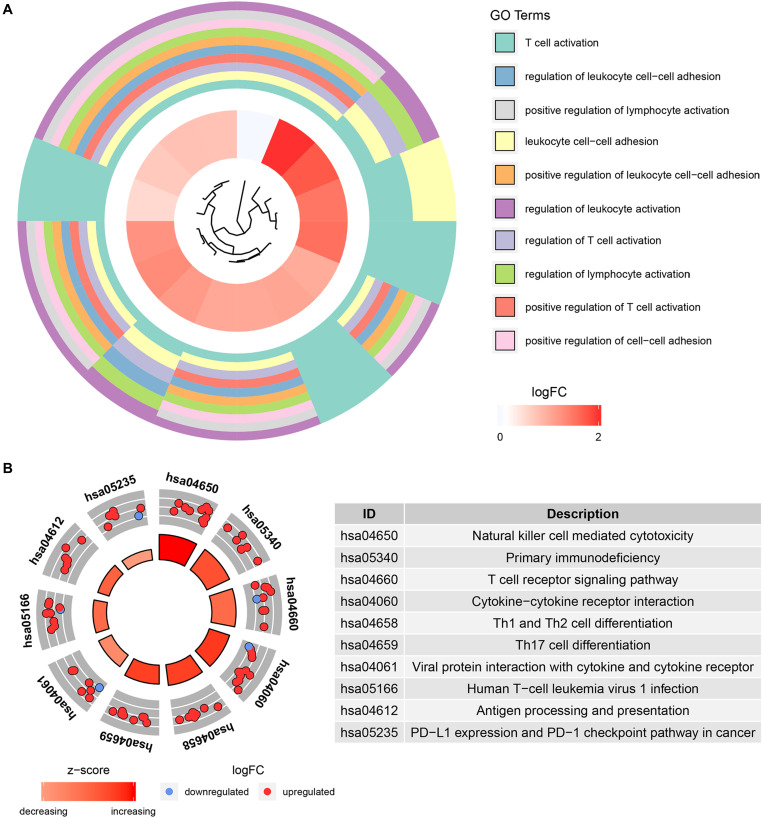
Bioinformatics analysis of genes based on 39 IRGs. **(A)** GO biological process analysis. **(B)** KEGG pathway enrichment.

To further understand the function of the 39 IRGs in hub modules, GO analysis was performed. IRGs in the relevant module were enriched in 258 BPs (Supplementary Table S2). KEGG analysis results showed that IRGs were significantly enriched in 28 BPs (Supplementary Table S3). The top 10 enriched BPs were T cell activation, regulation of leukocyte cell–cell adhesion, positive regulation of lymphocyte activation, leukocyte cell–cell adhesion, positive regulation of leukocyte cell–cell adhesion, regulation of leukocyte activation, regulation of T cell activation, regulation of lymphocyte activation, positive regulation of T cell activation, and positive regulation of cell–cell adhesion (Figure 6A). Moreover, KEGG pathway enrichment analysis results indicated that IRGs in the relevant module were involved in natural killer cell mediated cytotoxicity, primary immunodeficiency, T cell receptor signaling pathway, cytokine–cytokine receptor interaction, Th1 and Th2 cell differentiation, Th17 cell differentiation, viral protein interaction with cytokine and cytokine receptor, human T-cell leukemia virus 1 infection, antigen processing and presentation, PD-L1 expression, and PD-1 checkpoint pathway in cancer (Figure 6B).

### Hub Gene Validation

**FIGURE 7:**
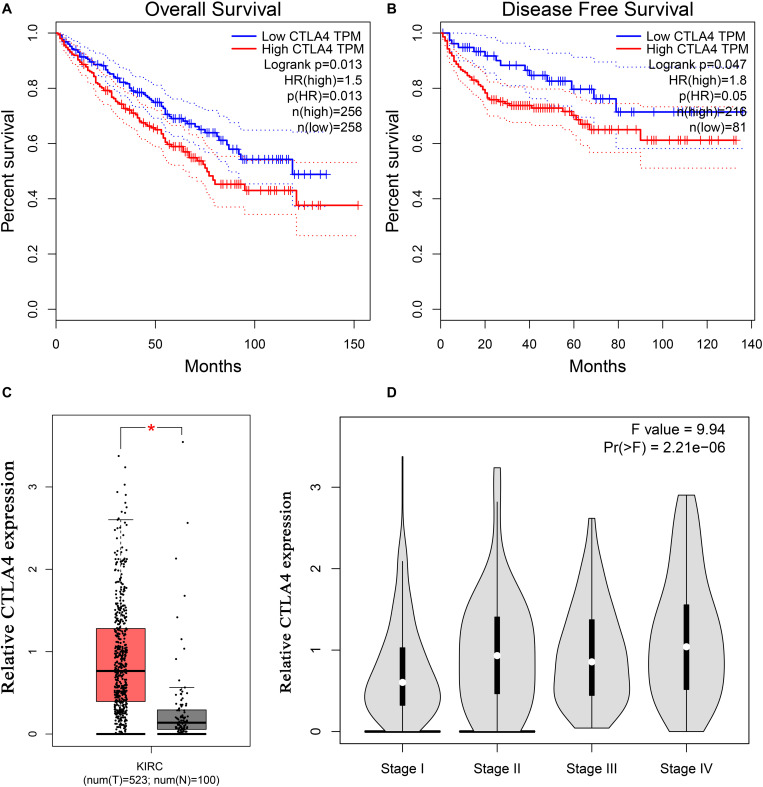
Validation of CTLA4. Kaplan–Meier survival curve based on the GEPIA database revealed that ccRCC patients with a higher expression of CTLA4 had a significantly shorter **(A)** overall survival time and **(B)** disease-free survival time. **(C)** Expressions of CTLA4 in ccRCC were significantly higher than those in normal tissues based on the TCGA-KIRC database (*P < 0.05). **(D)** High expression of CLTA4 related to higher tumor stage.

**FIGURE 8:**
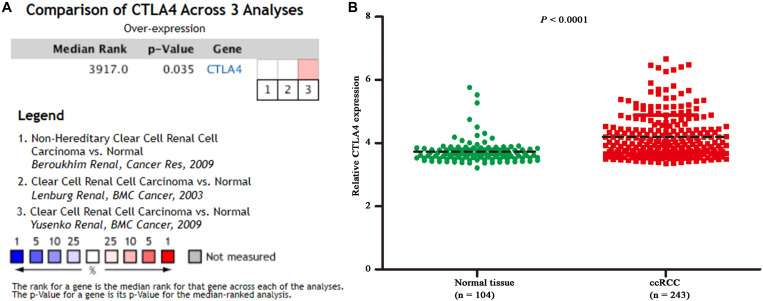
Oncomine and MMD database analyses. **(A)** Comparison of CTLA4 mRNA expression across 3 analyses of ccRCC based on Oncomine database. **(B)** Comparison of CTLA4 mRNA expression across 104 normal tissues and 243 cancerous tissues based on MMD database (*P* < 0.0001).

**FIGURE 9:**
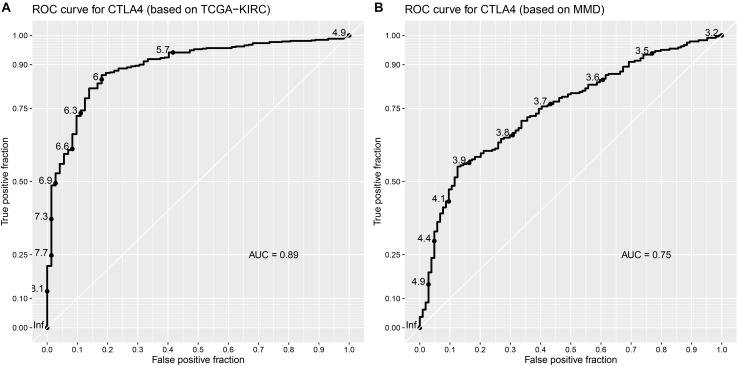
Validation of the prognostic value of CTLA4. **(A)** ROC curve for CTLA4 based on TCGA-KIRC (AUC = 0.89). **(B)** ROC curve for CTLA4 based on MMD (AUC = 0.75).

Based on the GEPIA database, patients with a higher expression of CTLA4 had a significantly shorter OS time (hazard ratio [HR] = 1.5, *P* = 0.013) and DFS time (HR = 1.8, *P* = 0.05) (Figures 7A,B). In addition, comparison of the mRNA expression levels of hub genes between tumors and normal tissues suggested that expression of CTLA4 in tumor tissues was significantly higher than the expression in normal tissues (*P* < 0.05) (Figure 7C). High expression of CLTA4 related to higher tumor stage (*F* = 9.94, *P* = 2.21E-06; Figure 7D). After that, we further compared CTLA4 expression levels between tumor tissues and normal tissues by using the Oncomine database for an external validation. The result suggested that the mRNA expression of CLTA4 was lower in normal tissues compared with ccRCC tissues (*P* = 0.035, Figure 8A). We also compared 104 normal tissues to 243 cancerous tissues from the MMD database and obtained the same conclusion (*P <* 0.0001) (Figure 8B). After the external and internal validation of the mRNA was completed, we validated the prognostic value of CTLA4. The ROC curve showed that CTLA4 exhibited excellent diagnostic efficiency for ccRCC (AUC = 0.89 and 0.75, respectively, Figures 9A,B) using the TCGA-KIRC and MMD databases.

### Correlation of CTLA4 Expression With Immune Infiltration Level in ccRCC

**FIGURE 10:**
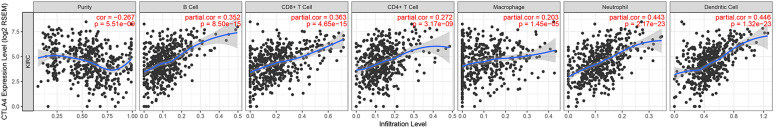
Correlation of CTLA4 expression with immune infiltration level in ccRCC.

Immune infiltration plays a significant role in tumor survival and progression. Therefore, we explored the relationship between hub genes and the level of immune infiltration according to the TIMER database. CTLA4 expression was positively related to dendritic cells (cor = 0.446, *P* = 1.32E-23) and negatively associated with tumor purity (cor = −0.267, *P* = 5.51E-09, Figure 10).

### CTLA4 Was Associated With Six Immune-Related Pathways

**FIGURE 11:**
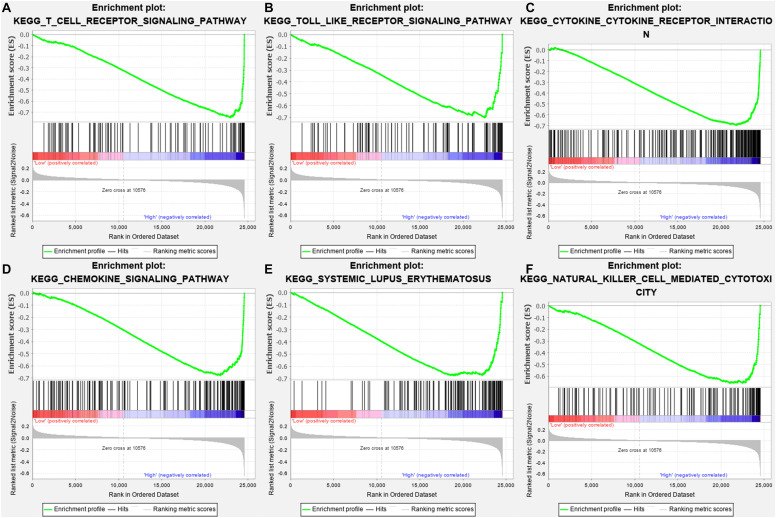
Gene-set enrichment analysis (GSEA) analysis for gene sets related with CTLA4 expression. **(A–F)** The gene sets of “T cell receptor signaling pathway,” “Toll like receptor signaling pathway,” “Cytokine–cytokine receptor interaction,” “Chemokine signaling pathway,” “Systemic lupus erythematosus,” and “Natural killer cell-mediated cytotoxicity” were enriched in ccRCC samples with CTLA4 highly expressed.

Gene-set enrichment analysis demonstrated that CTLA4 was significantly associated with six KEGG signaling pathways, including “T cell receptor signaling pathway” (nominal *P* = 0, | ES| = 0.747, gene size = 108 and FDR = 1.799%), “Toll-like receptor signaling pathway” (nominal *P* = 0.002, | ES| = 0.701, gene size = 101 and FDR = 1.772%), “Cytokine–cytokine receptor interaction” (nominal *P* = 0, | ES| = 0.693, gene size = 257 and FDR = 2.519%), “Chemokine signaling pathway” (nominal *P* = 0, | ES| = 0.679, gene size = 185 and FDR = 2.25%), “Systemic lupus erythematosus” (nominal *P* = 0.009, | ES| = 0.674, gene size = 129 and FDR = 4.589%), and “Natural killer cell mediated cytotoxicity” (nominal *P* = 0, |ES| = 0.658, gene size = 131 and FDR = 1.603%) (Table 1). Six functional gene sets were enriched on immune-related pathways (Figure 11).

**TABLE 1:** Genet-set enrichment analysis (GSEA) of CTLA4.

NAME	SIZE	ES	NES	NOM p-val	FDR
KEGG_T_CELL_RECEPTOR_SIGNALING_PATHWAY	108	−0.74698	−2.04543	0	0.01799
KEGG_TOLL_LIKE_RECEPTOR_SIGNALING_PATHWAY	101	−0.70073	−2.00497	0.001883	0.01772
KEGG_CYTOKINE_CYTOKINE_RECEPTOR_INTERACTION	257	−0.69304	−1.77987	0	0.025187
KEGG_CHEMOKINE_SIGNALING_PATHWAY	185	−0.67969	−1.8281	0	0.022468
KEGG_SYSTEMIC_LUPUS_ERYTHEMATOSUS	129	−0.67417	−1.70733	0.009901	0.045895
KEGG_NATURAL_KILLER_CELL_MEDIATED_CYTOTOXICITY	131	−0.65813	−1.89423	0	0.016028

### DEG Screening

**FIGURE 12:**
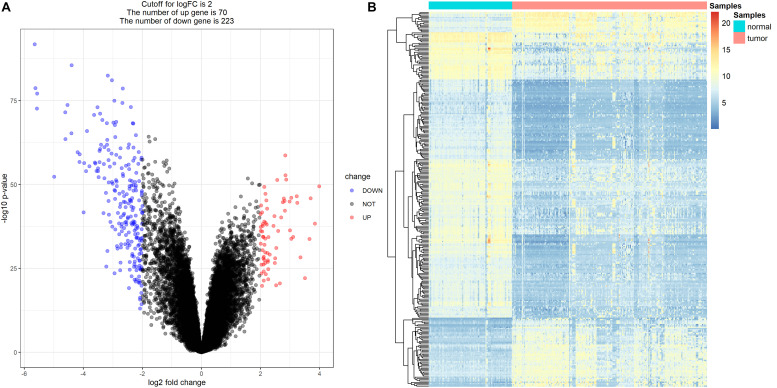
Differentially expressed genes (DEGs) screening. **(A)** Volcano plot visualizing the immune-related DEGs. **(B)** Heatmap for immune-related DEGs between tumor samples and normal samples (*P* < 0.05, fold change > 2).

Because drug exploration is based on DEGs, we first screened out DEGs. After data preprocessing, expression matrices were obtained from the 347 samples in the MMD dataset. A total of 293 DEGs (70 upregulated and 223 downregulated) were selected (Figures 12A,B). The adjusted *P*-value and log2FC of each immune-related DEG are detailed in Supplementary Table S4.

### Novel Choices for ccRCC Treatment

After the CMap was performed, a total of nine molecule drugs were screened out (Table 2). Among them, three small molecule drugs – monensin (|enrichment| = 0.865, *P* = 0.000), quercetin (|enrichment| = 0.614, *P* = 0.010), and fenbufen (|enrichment| = 0.599, *P* = 0.015) – might be novel choices for ccRCC treatment.

**TABLE 2:** Results of CMap analysis based on DEGs in ccRCC.

cmap name	Mean	n	Enrichment	p	Specificity	% non-null
Monensin	0.677	6	0.865	0.00002	0	100
Quercetin	0.288	6	0.614	0.01029	0.0107	50
Fenbufen	−0.321	6	−0.599	0.01458	0.0174	50
Karakoline	0.44	6	0.541	0.03601	0.0428	66
LY-294002	−0.345	61	−0.46	0	0.0859	59
Resveratrol	−0.411	9	−0.522	0.00819	0.1667	66
Rofecoxib	−0.296	6	−0.524	0.0471	0.0571	50
Helveticoside	−0.263	6	−0.556	0.02872	0.1429	50
6-Bromoindirubin-3’-oxime	−0.349	7	−0.583	0.00874	0.1122	57

*N: number of instances; enrichment: enrichment score; p: permutation p; specificity: the frequency at which the enrichment of a set of instances in the ordered list of all instances in a given result is equaled or exceeded; non-null percentage: the percentage of all instances in a set of instances that share the majority non-null category of connectivity score.*

## Discussion

For localized RCC, surgery is the only curative treatment that is supported by high-quality evidence, while systemic treatment is necessary for patients with metastatic RCC (Ljungberg et al., 2015). However, ccRCC is usually resistant to chemoradiotherapy. Targeted therapy may be the best choice of non-surgical treatments because of their target specificity and low toxicity (Vera-Badillo et al., 2015). Nevertheless, the intra-tumor molecular heterogeneity of ccRCC may influence the response to targeted therapy (Hong et al., 2017). Resistance to targeted therapies is also a major problem (Holohan et al., 2013). The prognosis for patients with metastatic RCC remains poor despite systemic therapy. Early diagnosis with individualized treatment and follow-up is the key to successful outcomes. The identification of more effective biomarkers and immunotherapeutic targets for ccRCC is an urgent goal.

Immunotherapy is emerging as a new treatment for ccRCC. The long-term use of endocrine therapy and targeted biotherapy has increased the understanding of the immune escape of cancer cells, and the discovery of selective immune checkpoints has created new opportunities for treatment. Many articles have focused on the discovery of immune-related prognostic biomarkers and therapeutic targets for cancer. Ito et al. (2018) reported that the mRNA levels of the IRGs PD-L1 and CD8 may reflect the antitumor immune response, with low PD-1 and high PD-L1 mRNA levels independently implicated as poor prognostic markers in gastric cancer patients who underwent surgery. Bai et al. (2019) reported the involvement of seven IRGs in the occurrence, development, malignant transformation, and pathology of breast cancer. Therefore, immune-related prognostic biomarkers are highly correlated with cancer progression and prognosis. However, similar data regarding ccRCC remains scarce.

In this study, we identified 39 hub genes by constructing a co-expression network for IRGs (TCGA-KIRC). GO and KEGG database analyses revealed that they were enriched on immune-related pathways. A PPI network further demonstrated that CTLA4 had the highest connectivity degree among the identified genes. CTLA4 was validated as being closely correlated with the estimated clinical trait.

CTLA-4 (Zhao et al., 2018) is a membrane glycoprotein expressed by activated effector T cells involved in inhibition of T cell proliferation. Although CTLA4 is expressed on both CD4 and CD8 lymphocytes, it plays a significant role in adjusting the activity of CD4-positive cells. CTLA4 can enhance the inhibitory effect of T regulatory cells and decrease the activity of T helper cells (Carosella et al., 2015). CTLA4 also plays an important role in cancer progression, prognosis, and proliferation. Overexpression of CTLA-4 by lymphocyte subsets might be closely correlated with lung cancer (Erfani et al., 2012). On the other hand, a high CTLA4 mRNA level was associated with breast cancer patients having higher clinical staging and lymph node metastasis (Mao et al., 2010). CTLA4 overexpression was also found to be a positive prognostic marker in nasopharyngeal cancer and malignant pleural mesothelioma (Huang et al., 2016; Roncella et al., 2016). Therefore, we further explored the potential functions of CTLA4.

As an IRG, CTLA4 was overexpressed in ccRCC tissues, compared with normal renal tissues. Based on the GEPIA database, we found that patients with a higher expression of CTLA4 had shorter OS time and DFS time. In addition, the expression of CTLA4 increased with the progression of ccRCC. Analyses involving the Oncomine database and MMD database suggested that the mRNA expression of CLTA4 was higher in ccRCCs compared with normal tissues. The findings support the view that CTLA4 is crucial in the progression of ccRCC and may be a novel immune-related prognosis biomarker.

Considering that the immune infiltration level has been strongly correlated with survival in tumors, we studied the relationship between CTLA4 expression and immune infiltration level in ccRCC using the TIMER database. CTLA4 expression was positively related to dendritic cells and negatively associated with tumor purity, indicating that CTLA4 has significant roles in immune infiltration cells in ccRCC.

We also explored some novel choices for ccRCC treatment. First, 293 DEGs were obtained by integrating six data sets of the GEO database. CMap analysis was then carried out. Three small molecule drugs (monensin, quercetin, and fenbufen) showed strong potential for ccRCC treatment.

There had been some limitations in this study. Although we designed this bioinformatic study well and used strict thresholds for each database mining and subsequent analysis, the major drawback in this study was the lack of novelty. We did not find relevant data for the verification of protein expression of CTLA4 based on the Human Protein Atlas database (Uhlen et al., 2015)^9^. On the other hand, though we used strict thresholds for each part in our study, there was no external experimental verification. Related mechanisms of CTLA4 in ccRCC will be explored *in vivo* and *in vitro* in further analyses. We also will further evaluate the potential of the proposed small molecular drugs in the short future.

## Conclusion

We identified 39 hub genes by constructing co-expression network for IRGs and identified and validated network hub genes associated with the progression and poor prognosis of ccRCC. CTLA4 was identified and validated as being associated with the progression and poor prognosis of ccRCC. Three molecule drugs (monensin, quercetin, and fenbufen) were identified and may be novel choices for ccRCC treatment. Our study could provide novel immune-related targets for studies of the pathogenesis of ccRCC and potential new immunotherapy drugs for the treatment of ccRCC.

## Data Availability Statement

The data that support the findings of this study are openly available in Gene Expression Omnibus (GEO) database at http://www.ncbi.nlm.nih.gov/geo/ (GSE53757, GSE11151, GSE12090, GSE12606, GSE23629, and GSE36895), and The Cancer Genome Atlas (TCGA) database at https://genomecancer.ucsc.edu/(ccRCC).

## Author Contributions

W-LH, G-FX, and XY conceived and designed the study. G-FX and XY conducted all analysis procedures. G-FX, XY, ZC, R-JZ, T-ZL, and W-LH analyzed the results. W-LH, T-ZL, and XY contributed the analysis tools. G-FX contributed to the writing of the manuscript. All authors reviewed and approved the manuscript.

## Conflict of Interest

The authors declare that the research was conducted in the absence of any commercial or financial relationships that could be construed as a potential conflict of interest.

## Footnotes

1 http://www.ncbi.nlm.nih.gov/geo/

2 https://genomecancer.ucsc.edu/

3 https://www.immport.org

4 http://string.embl.de/

5 http://gepia.cancer-pku.cn/

6 https://www.oncomine.org/

7 https://cistrome.shinyapps.io/timer/

8 http://software.broadinstitute.org/gsea/index.jsp

9 http://www.proteinatlas.org

## Supplementary Material

FIGURE S1 Determination of soft-thresholding power in the weighted gene co-expression network analysis (WGCNA). **(A)** Analysis of the scale-free fit index for various soft-thresholding powers (β). **(B)** Analysis of the mean connectivity for various soft-thresholding powers. **(C)** Histogram of connectivity distribution when β = 4. **(D)** Checking the scale free topology when β = 4.

TABLE S1 Degree of connectivity of hub genes in the co-expression network (TCGA-KIRC).

TABLE S2 GO biological processes of hub genes in the co-expression network.

TABLE S3 KEGG enrichment of hub genes in the co-expression network.

TABLE S4 Differentially expressed genes (DEGs) between normal samples and ccRCC samples (based on MMD).
