# Supplementary figures and images for: Identification of a Novel Immune-Related Prognostic Biomarker and Small-Molecule Drugs in Clear Cell Renal Cell Carcinoma (ccRCC) by a Merged Microarray-Acquired Dataset and TCGA Database
Source: Front Genet. 2020 Aug 18;11:810. doi: 10.3389/fgene.2020.00810 (PMC7461880; doi:10.3389/fgene.2020.00810)

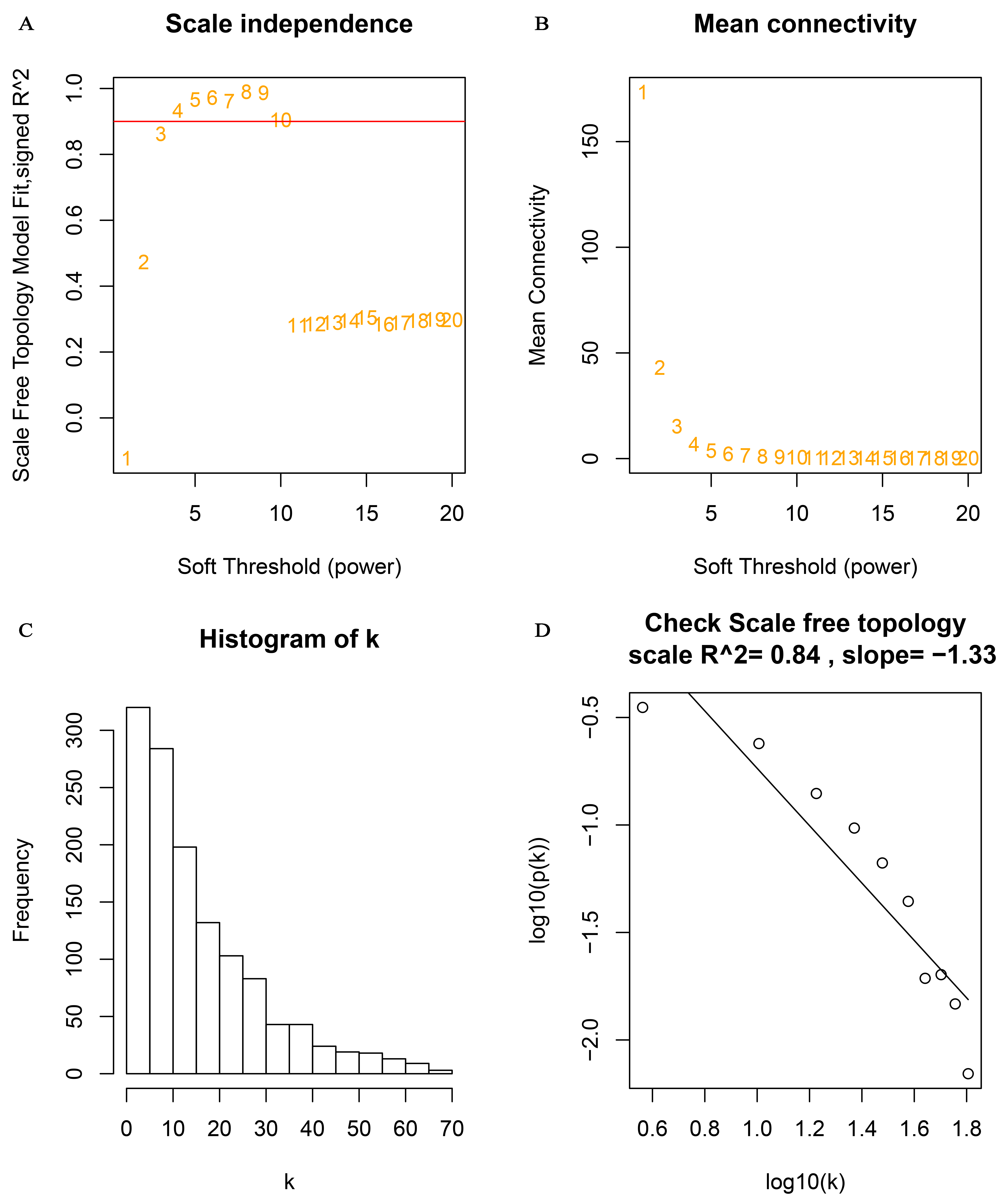

Supplement: FIGURE S1 — Determination of soft-thresholding power in the weighted gene co-expression network analysis (WGCNA). (A) Analysis of the scale-free fit index for various soft-thresholding powers (β). (B) Analysis of the mean connectivity for various soft-thresholding powers. (C) Histogram of connectivity distribution when β = 4. (D) Checking the scale free topology when β = 4. [file Image_1.TIF]
